# Pica in Child Psychiatry: Clinical Profile, Associated Factors and Management

**DOI:** 10.1192/j.eurpsy.2026.11172

**Published:** 2026-07-31

**Authors:** R. Bendada, Z. El Maataoui

**Affiliations:** Hospital Arrazi, Salé, Morocco

## Abstract

**Introduction:**

Pica is an eating behavior disorder characterized by the persistent ingestion of non-nutritive, non-food substances. Although rare, it often remains underdiagnosed in child psychiatry. It may occur in the context of neurodevelopmental disorders, nutritional deficiencies, or psychosocial vulnerability. Early identification is essential to prevent both medical and psychological complications.

**Objectives:**

To describe the clinical profile, associated factors, and management strategies of two pediatric cases of Pica, and to discuss these findings in light of current literature.

**Methods:**

We report two clinical observations of children followed in the Child Psychiatry Department of Ar-Razi University Hospital in Salé, Morocco. This descriptive and qualitative study is based on a detailed analysis of clinical, developmental, and family data, complemented by a literature review. Each case was assessed using a multidisciplinary approach including psychiatric, psychological, biological, and educational evaluations.

**Clinical observations:** *Case 1 – Rayan (9 years old):* A boy living in a conflictual family environment, presenting since kindergarten with ingestion of soil, paper, and wall fragments, associated with ADHD, language disorder, learning difficulties, and separation anxiety. *Case 2 – El Mehdi (9 years old):* A boy from a rural background, showing hair and thread ingestion (trichophagia) since age 2, complicated by gastric trichobezoars requiring two surgeries, within a context of prolonged maternal separation and persistent nocturnal enuresis.

**Results:**

These two cases highlight the clinical heterogeneity of Pica, which often overlaps with neurodevelopmental disorders, anxiety symptoms, or insecure attachment patterns. Current literature supports a multifactorial etiology involving biological, psychological, and environmental components. Early recognition and comprehensive evaluation are crucial for appropriate intervention.

**Conclusions:**

Although uncommon, Pica should be systematically screened in child psychiatry. A multidisciplinary management approach — combining psychoeducation, behavioral interventions, nutritional monitoring, and family support — contributes to improved prognosis and child well-being.

**Disclosure of Interest:**

None Declared

